# Comprehensive consideration of multiple determinants from evidence to recommendations in guidelines for most traditional Chinese medicine was suboptimal: a systematic review

**DOI:** 10.1186/s12906-023-04321-0

**Published:** 2024-01-04

**Authors:** Yi-Cheng Gao, Rui Cao, Zhi-Han Liu, Ying-Di Liao, Li-Yuan Tao, Yu-Ting Feng, Qian-Yun Chai, Min-Jing Luo, Yu-Tong Fei

**Affiliations:** 1 Centre for Evidence-Based Chinese Medicine, Beijing University of Chinese Medicine, Beijing, China; 2 Institute of Excellence in Evidence-Based Chinese Medicine, Beijing University of Chinese Medicine, Beijing, China; 3 Beijing GRADE Centre, Beijing, China; 4 Kunming Traditional Chinese Medicine Hospital, Kunming, China; 5 Research Center of Clinical Epidemiology, Peking University Third Hospital, Beijing, China

**Keywords:** Clinical practice guidelines, Evidence to recommendation, Discordant recommendations, Inappropriate discordant recommendations, Traditional Chinese Medicine

## Abstract

**Background:**

The overall comprehensive consideration of the factors influencing the recommendations in the traditional Chinese medicine (TCM) guidelines remains poorly studied. This study systematically evaluate the factors influencing recommendations formation in the Grading of Recommendations Assessment, Development, and Evaluation (GRADE) clinical practice guidelines (CPGs) and TCM CPGs.

**Methods:**

This was a methodological review in which we searched six databases and multiple related websites. The GRADE CPGs were identified as the guidelines developed by the GRADE Working Group or the two Co-Chairs. For the TCM CPGs, we randomly selected guidelines that were published by the TCM or integrative medicine academic societies from China mainland (published by the TCM or integrative medicine academic societies of China mainland). Two reviewers independently screened and extracted data. We included CPGs published in 2018–2022. We extracted information on the influencing factors of evidence to recommendation and conducted the analyses using descriptive statistics and calculated the proportion of relevant items by IBM SPSS Statistics and Microsoft Excel to compare the differences between the GRADE CPGs and the TCM CPGs.

**Results:**

Forty-five GRADE CPGs (including 912 recommendations) and 88 TCM CPGs (including 2452 recommendations) were included. TCM recommendations mainly considered the four key determinants of desirable anticipated effects, undesirable anticipated effects, balance between desirable and undesirable effects, certainty of evidence, with less than 20% of other dimensions. And TCM CPGs presented more strong recommendations (for or against) and inappropriate discordant recommendations than GRADE CPGs. GRADE CPGs were more comprehensive considered about the factors affecting the recommendations, and considered more than 70% of all factors in the evidence to recommendation.

**Conclusions:**

The TCM CPGs lack a comprehensive consideration of multiple influencing determinants from evidence to recommendations. In the future, the correct application of the GRADE approaches should be emphasized.

**Supplementary Information:**

The online version contains supplementary material available at 10.1186/s12906-023-04321-0.

## Introduction

Traditional Chinese medicine (TCM) includes acupuncture, massage, formula, Chinese patent drug, etc., especially acupuncture, is a widely used complementary and alternative therapy [1–4]. The application of TCM therapy in clinical practice requires more assertive guidance. TCM clinical practice guidelines (CPGs) play a huge role as guidelines for recommendations for TCM intervention. At present, several authoritative Traditional Chinese Medicine societies in China have published a considerable number of clinical practice guidelines to assist clinicians in making decisions [5–11].

Reliable, trustworthy CPGs, based on systematic evidence review and comprehensive consideration of various factors influencing the recommendations, are important as an guidance document for clinicians’ practice [12]. The Grading of Recommendations Assessment, Development, and Evaluation (GRADE) approaches is currently recognized as an international gold standard by various organizations worldwide and greatly improving the overall quality of guidelines development [13–17]. In general, The GRADE CPGs developed by the GRADE Working Group or the two Co-Chair, professors Gordon H. Guyatt and Holger J. Schünemann will strictly implement the GRADE approach. The GRADE approaches comprehensively considered the multiple factors that affect the recommendations (such as desirable anticipated effects, undesirable anticipated effects, certainty of evidence, etc.) through standard, structured and transparent methods, and avoids the increased implementation difficulty or being questioned of the CPGs caused by incomplete consideration [18]. Additionally, certainty of evidence, as one of the key determinants affecting the formation of recommendations, is clear associated with the strength of recommendations [19, 20]. Respecting the relationship between the two can avoid misleading as much as possible [21].

There were many studies on the methodological quality of TCM CPGs [22–24], but they focused more on the overall quality and content of the guidelines. The methodological on the influencing factors of the formation of evidence to recommendations remain largely under-explored. The systematic search of this study was conducted and critically evaluated the consideration of the GRADE CPGs and TCM CPGs on the factors influencing the recommendations, and compared the differences and objective gap between the two.

## Methods

### Literature search

Two reviewers searched databases including PubMed, Embase, China National Knowledge Infrastructure, VIP Database for Chinese Technical Periodicals, Chinese Biomedical Literature Database, and Wanfang (The full database search strategy is depicted in Additional file 1: Appendix A).

For the GRADE CPGs, we also searched the https://www.GRADEpro.org/ website as well as the methodological literature published by the two co-chairs and screened through the guidelines mentioned in those literature. For the TCM CPGs, We also searched six authoritative TCM society websites, including China Association of Chinese Medicine, China Association of Traditional Chinese Medicine, Chinese Association of Integrative Medicine, China Association for Acupuncture and Moxibustion, World federation of Chinese medicine societies, Doctor Society of integrative Medicine, Chinese Medical Doctor Association.

### Eligibility criteria of CPGs

This study included GRADE CPGs and TCM CPGs published from 1 January 2018 to 31 December 2022. We consulted several guideline development methodology experts and used focus group discussions to determine the selection of GRADE CPGs and TCM CPGs. The GRADE CPGs were identified as the guidelines developed by the GRADE Working Group or the two Co-Chair, professors Gordon H. Guyatt and Holger J. Schünemann. For the TCM CPGs, We adopted simple random sampling, and randomly selected 15 of the guidelines published by six authoritative TCM societies and other societies, and less than 15 were all included. We excluded repeated publication as well as older versions of the CPGs.

### Data extraction

Reviewers independently screened all titles, abstracts, full texts as well as data extraction, and discrepancies were resolved through consultation or by a third author (FY). Two kinds of information were extracted: (1) The basic characteristics of the CPGs, including the CPGs type, scope, discipline, whether the GRADE approaches were adopted, whether the certainty of evidence and the strength and direction of recommendations were reported, (2) relevant information on the influencing factors of evidence to recommendation, including the factors considered when the formation of recommendation (desirable or undesirable anticipated effects, certainty of the evidence, values and preferences, health equity and other factors) [15, 17], whether it is supported by evidence, whether it is considered as an independent dimension, the association between the certainty of evidence and the strength of recommendations, and the number of discordant and inappropriate discordant recommendations.

We defined discordant recommendations as strong recommendations based on low or very low certainty of evidence [25]. There are two types of discordant recommendations, appropriate or inappropriate. There are five special conditions that were allowed for strong recommendations based on low or very low certainty evidence: life-threatening situation is beneficial; Uncertain evidence is beneficial but high certainty of evidence is harmful; Low or very low certainty evidence has equal benefits, high-certainty evidence suggests that one is less harmful; High-certainty of evidence has equal benefits, and low or very low of certainty of evidence suggests that one of them is more harmful; Uncertain benefits but with potential catastrophic harm. Discordant recommendations that were made under one of the five special conditions were considered as appropriate, otherwise, inappropriate [26].

### Data analysis

This study conducted the analyses using descriptive statistics and calculating the proportion of relevant items to compare the differences between the GRADE CPGs and the TCM CPGs. For recommendations access certainty of evidence beyond the GRADE approaches used, we consider the highest level as high-certainty, the second high level as moderate-certainty, the third high level as low-certainty, and the other levels as very-low-certainty. For recommendations used criteria other than the GRADE approaches to present the strength of recommendations, we consider that the explicit expression of recommendation strength is strong, or recommendations based on the multiple level of recommendation, the highest level is strong recommend or against, the others are conditional (Additional file 2: Appendix B).

## Results

### Search results

**Fig. 1:**
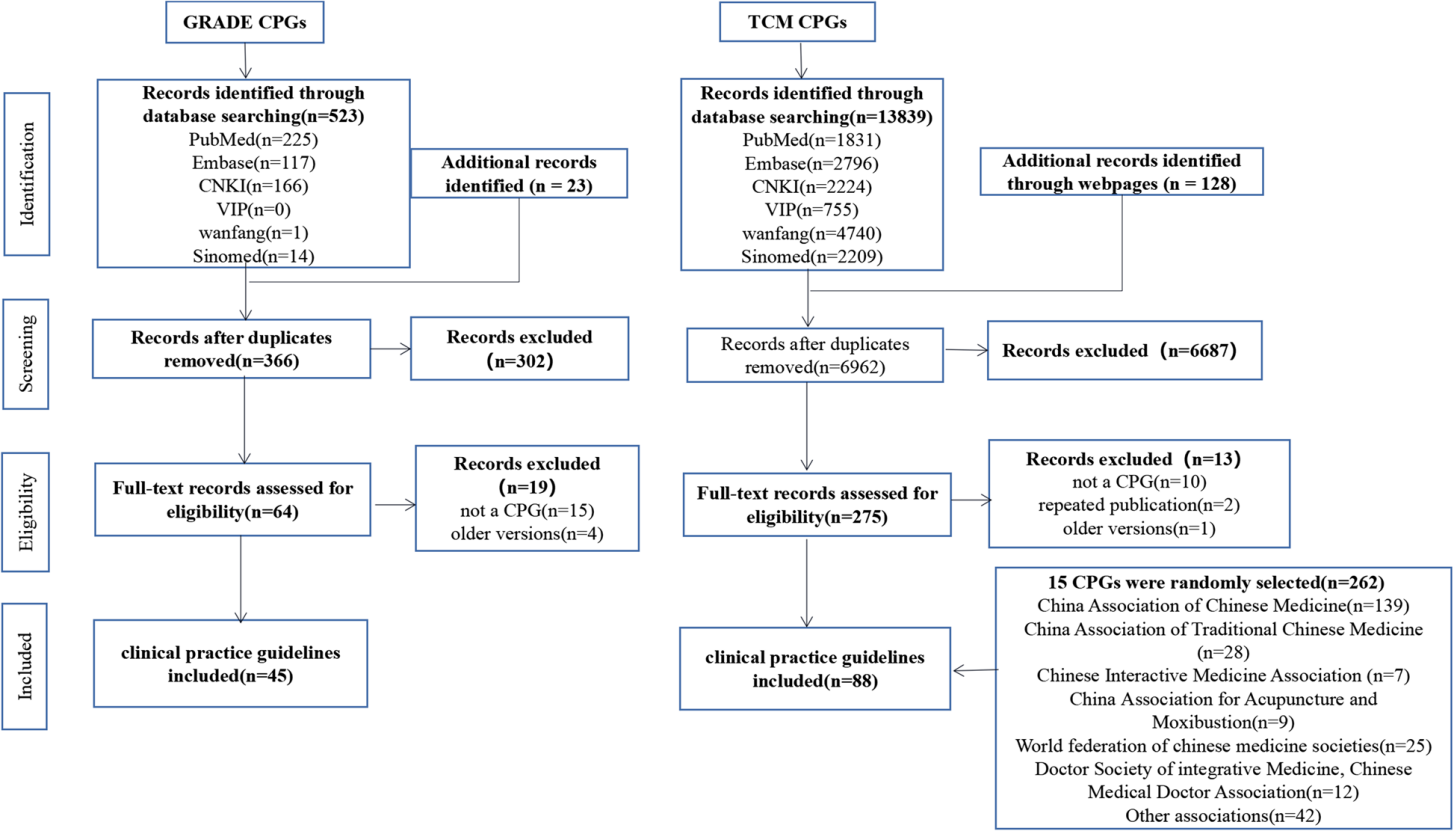
Flow chart of selecting clinical practice guidelines

The GRADE CPGs and the TCM CPGs were searched separately. For the GRADE CPGs, a total of 523 records were identified through the database search, and 23 from other sources, and 180 duplicates were excluded. 302 were deemed ineligible, with 64 records remaining. We excluded 19 for the following reasons: 15 were not a CPG, and 4 were old version, and finally included 45 CPGs. For the TCM CPGs, a total of 13839 records were identified through the database search, and 128 from other sources, and 6962 remained after excluding duplicates, of those, 6697 were deemed ineligible, 13 CPGs were excluded for the following reasons: 10 were not a CPG, and 1 was old version, 2 were duplicate publications, and finally, We included 88 of the 262 TCM CPGs by simple random sampling (The details of the included 133 CPGs were displayed in Additional file 3: Appendix C and the screening process was detailed in Fig. 1).

### Characteristics of included CPGs

Forty-five were GRADE CPGs, 262 were TCM CPGs. All GRADE CPGs used GRADE approaches to develop guidelines and all report certainty of evidence and strength and direction of recommendation. 131 (50.0%) TCM CPGs did not use GRADE approaches, 35 (13.4%) CPGs did not report certainty of evidence and strength and direction of recommendation. The detailed characteristics of CPGs were summarized in the Table 1.

**Table 1:** Characteristic of included clinical practice guidelines N(%)

The baseline characteristics of guidelines	GRADE CPGs^a^ (*n* = 45)	TCM CPGs^b^ (*n* = 262)	Total^c^ (*n* = 307)
**Type of guidelines**			
Routine evidence-based guidelines	26(57.8)	234(89.3)	260(84.7)
Rapid recommendations	16(35.6)	0(0.0)	16(5.2)
Adaptation guideline	2(4.4)	6(2.3)	8(2.6)
Living guideline	1(2.2)	0(0.0)	1(0.3)
Non-evidence-based guideline	0(0.0)	22(8.4)	22(7.2)
**Scope of guidelines**			
Treatment	19(42.2)	118(45.0)	137(44.6)
Diagnosis	1(2.2)	0(0.0)	1(0.3)
Management^d^	13(28.9)	7(2.7)	20(6.5)
Treatment and diagnosis	1(2.2)	137(52.3)^e^	138(45.0)
Prevention	6(13.3)	0(0.0)	6(2.0)
Screening	3(6.7)	0(0.0)	3(1.0)
Others^f^	2(4.4)	0(0.0)	2(0.7)
**Discipline**			
Internal medicine	32(71.1)	157(59..9)	189(61.6)
Surgery	3(6.7)	54(20.6)	57(18.6)
Gynaecology or Andrology	1(2.2)	25(9.5)	26(8.5)
Paediatrics	1(2.2)	18(6.9)	19(6.2)
COVID-19	5(11.1)	3(1.1)	8(2.6)
Others^g^	3(6.7)	5(1.9)	8(2.6)
**What to access using the GRADE approaches**			
Certainty of evidence and strength and direction of the recommendation	45(100.0)	105(40.1)	150(48.9)
Certainty of evidence	0(0.0)	14(5.3)	14(4.6)
Strength and direction of the recommendation	0(0.0)	12(4.6)	12(3.9)
Not applied	0(0.0)	131(50.0)	131(42.7)
**Report certainty of evidence and strength and direction of the recommendations**			
Report certainty of evidence and strength and direction of the recommendations	45(100.0)	202(77.1)	247(80.5)
Only report the certainty of evidence	0(0.0)	5(1.9)	5(1.6)
Only report the strength and direction of the recommendations	0(0.0)	20(7.6)	20(6.5)
Neither reported	0(0.0)	35(13.4)	35(11.4)

*CPG* clinical practice guidelines, *GRADE* Grading of Recommendations Assessment, Development and Evaluation, *TCM* Traditional Chinese Medicine

^a^The proportion of multiple items was compared to all 45 GRADE CPGs

^b^The proportion of multiple items was compared to all 262 TCM CPGs

^c^The proportion of multiple items was compared to all 307 CPGs

^d^It includes at least 2 kinds of recommendations in diagnosis, treatment, prevention, screening, detection, prognosis and life management

^e^The title of the guideline is defined as the treatment and diagnosis, but the diagnosis is the introduction of background information, and the recommendation is unrelated to the diagnosis

^f^For the application of the interventions: Transfusion strategies, lumbar puncture

^g^Transfusion strategies, Ebola virus disease, lumbar puncture, stomatology, application of drugs

### Association between number of GRADE factors adopted and strength and direction of recommendations

A total of 912 recommendations were presented from the 45 included GRADE CPGs. In the process of recommendation formation, all (912, 100%) five determinants, including desirable anticipated effects, undesirable anticipated effects, balance between desirable and undesirable effects, certainty of evidence and values and preferences, were considered, and almost all of them were considered as independent dimensions.

Less than 60% of the other factors were considered. It is worth noting that the CPGs published by the American College of Rheumatology considered other factors (eg, resource requirements, Health equity, acceptability, feasibility) poorly, even though they claim to use the GRADE approaches, excluding the recommendations in the eight American College of Rheumatology CPGs, the remaining GRADE recommendations considered more than 70% of all factors in the evidence to the decision (EtD) framework. However, TCM recommendations only considered the four determinants of desirable anticipated effects, undesirable anticipated effects, balance between desirable and undesirable effects, certainty of evidence, with less than 20% of other factors, and lack evidence support (Tables 2 and 3).

**Table 2:** Association between number of GRADE factors adopted and strength and direction of recommendations of the included CPGs N (%)

GRADE domain	GRADE recommendations (*n* = 912)			TCM recommendations (*n* = 2452)		
	**Considered**^**a**^	**Considered and supported by evidence**^**a**^	**Considered as an independent dimension**^**a**^	**Considered**^b^	**Considered and supported by evidence**^b^	**Considered as an independent dimension**^b^
Problem priority	426(46.7)	312(34.2)	426(46.7)	0(0.0)	0(0.0)	0(0.0)
Desirable anticipated effects	912(100.0)	842(92.3)	912(100.0)	2432(99.2)	2022(82.5)	2432(99.2)
Undesirable anticipated effects	912(100.0)	842(92.3)	912(100.0)	2432(99.2)	2022(82.5)	2432(99.2)
Balance between desirable and undesirable effects	912(100.0)	842(92.3)	912(100.0)	2432(99.2)	2022(82.5)	2432(99.2)
Certainty of the evidence	912(100.0)	842(92.3)	912(100.0)	2394(97.6)	2000(81.6)	2394(97.6)
Values and preferences	912(100.0)	353(38.7)	907(99.5)	403(16.4)	4(0.2)	403(16.4)
Resource requirements	515(56.5)	393(43.1)	465(51.0)	475(19.4)	6(0.2)	475(19.4)
Certainty of the evidence of resource requirements	359(39.4)	245(26.9)	358(39.3)	0(0.0)	0(0.0)	0(0.0)
Cost-effectiveness of the intervention	433(47.5)	202(22.1)	429(47.0)	2(0.1)	2(0.1)	2(0.1)
Health equity	466(51.1)	145(15.9)	431(47.3)	7(0.3)	2(0.1)	0(0.0)
Acceptability	493(54.1)	163(17.9)	431(47.3)	73(3.0)	0(0.0)	70(2.9)
Feasibility	496(54.4)	187(20.5)	436(47.8)	81(3.3)	4(0.2)	74(3.0)
Others^c^	37(4.1)	36(3.9)	30(3.3)	28(1.1)	4(0.2)	21(0.9)

*GRADE* Grading of Recommendations Assessment, Development and Evaluation, *TCM* Traditional Chinese Medicine

^a^The proportion of multiple items was compared to all 912 GRADE recommendations

^b^The proportion of multiple items was compared to all 2452 TCM recommendations

^c^human rights, policy, Regional compliance with the situation, the gap in the required clinical conditions and the practice, diagnostic accuracy, certainty of evidence for diagnostic accuracy, certainty of evidence for test effects, certainty of evidence of the management effect, certainty of evidence for diagnostic outcomes and management

**Table 3:** GRADE Recommendations published by the American College of Rheumatology or not N (%)

GRADE domain	GRADE recommendations	
	**Recommendations published by the American College of Rheumatology**^**a**^ **(*n* = 426)**	**Other GRADE guideline recommendations**^**b**^ **(*n* = 486)**
Problem priority	0(0.0)	426(87.7)
Desirable anticipated effects	426(100.0)	486(100.0)
Undesirable anticipated effects	426(100.0)	486(100.0)
Balance between desirable and undesirable effects	426(100.0)	486(100.0)
Certainty of the evidence	426(100.0)	486(100.0)
Values and preferences	426(100.0)	486(100.0)
Resource requirements	41(9.6)	474(97.5)
Certainty of the evidence of resource requirements	1(0.2)	358(73.7)
Cost-effectiveness of the intervention	1(0.2)	432(88.9)
Health equity	9(2.1)	457(94.0)
Acceptability	18(4.2)	475(97.7)
Feasibility	24(5.6)	472(97.1)
Others	0(0.0)	37(7.6)

*GRADE* Grading of Recommendations Assessment, Development and Evaluation

^a^The proportion of multiple items was compared to all 426 recommendations published by the American College of Rheumatology

^b^The proportion of multiple items was compared to all 486 remaining recommendations published by the American College of Rheumatology were excluded

**Fig. 2:**
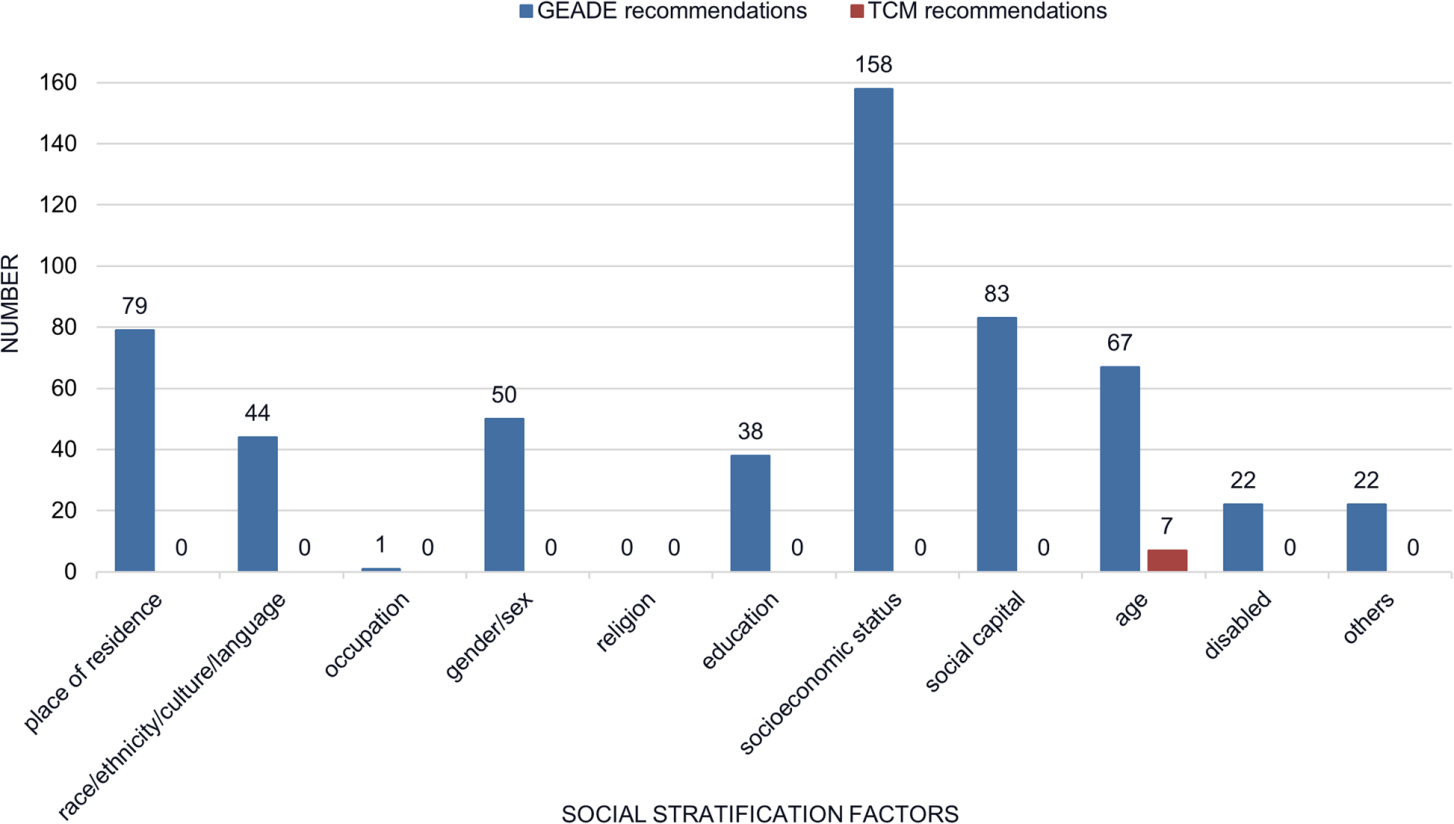
Presentation of social stratification factors in health equity in clinical practice guidelines

An additional aspect to note is that the GRADE CPGs performed a more detailed presentation of the factors considered. For instance, of the 466 GRADE recommendations considering health equity, 336 were presented in detail, including socioeconomic status (158, 46.2%), followed by social capital (83, 24.2%), age (67, 19.5%) and gender (50, 14.7%). However, the seven TCM recommendations were not, they all considered only the age (Fig. 2).

### Association between certainty of evidence and recommendations

Of the 912 GRADE recommendations, 864 reported the certainty of evidence and the strength and direction of the recommendations. Of the 2,452 TCM recommendations, 2,094 reported the certainty of evidence and the strength and direction of the recommendations. The GRADE CPGs presented a large number of recommendations (191,22.1%) with strong against or conditional against, Conversely, there was only one (1, 0.0%) against recommendation in the TCM recommendations. Quite interestingly, we also found that the TCM CPGs presented more strong recommend or against recommendations than the GRADE guidelines (Table 4).

**Table 4:** Association between certainty of evidence and recommendation N (%)

Strength and direction of recommendations	high certainty of evidence	moderate certainty of evidence	low certainty of evidence	very low certainty of evidence	Total
**GRADE CPGs**					
Strong recommend	8(0.9)	59(6.8)	29(3.4)	52(6.0)	148(17.1)
Strong against	2(0.2)	11(1.3)	6(0.7)	17(2.0)	36(4.2)
Conditional recommend	3(0.3)	70(8.1)	155(17.9)	297(34.4)	525(60.8)
Conditional against	2(0.2)	9(1.0)	36(4.2)	108(12.5)	155(17.9)
Total	15(1.7)	149(17.2)	226(26.2)	474(54.9)	864(100.0)
**TCM CPGs**					
Strong recommend	94(4.5)	327(15.6)	222(10.6)	282(13.5)	925(44.2)
Strong against	0(0.0)	1(0.0)	0(0.0)	0(0.0)	1(0.0)
Conditional recommend	10(0.5)	138(6.6)	298(14.2)	721(34.4)	1167(55.7)
Conditional against	0(0.0)	1(0.0)	0(0.0)	0(0.0)	1(0.0)
Total	104(5.0)	467(22.3)	520(24.8)	1003(47.9)	2094(100)

*CPG* clinical practice guidelines, *GRADE* Grading of Recommendations Assessment, Development and Evaluation, *TCM* Traditional Chinese Medicine

### Discordant and inappropriate discordant recommendations

One hundred four discordant recommendations were identified in the GRADE CPGs, 91 (87.5%) were appropriate discordant recommendations. We identified 504 discordant recommendations in the TCM CPGs, but only 19 (3.8%) are appropriate discordant recommendations (Table 5).

**Table 5:** Appropriateness of recommendations with low or very low certainty of evidence N (%)

Discordant recommendations	Type of recommendations	
	**GRADE recommendations**^**a**^ **(*N* = 104)**	**TCM recommendations**^**b**^ **(*N* = 504)**
Appropriate discordant recommendations	91(87.5)	19(3.8)
Inappropriate discordant recommendations	13(12.5)	485(96.2)
Totals	104(100.0)	504(100.0)

*CPG* clinical practice guidelines, *GRADE* Grading of Recommendations Assessment, Development and Evaluation, *TCM* traditional chinese medicine

^a^The proportion of multiple items was compared to all 104 GRADE recommendations

^b^The proportion of multiple items was compared to all 504 TCM recommendations

## Discussion

### Summary of findings

Forty five GRADE CPGs (including 912 recommendations) and 88 TCM CPGs (including 2452 recommendations) were included in this study. Compared with the GRADE CPGs, half of the TCM CPGs did not apply GRADE approaches, and the TCM recommendations have relatively insufficient consideration of some factors, such as values and preferences, health equity, resource requirements, and evidence to support them was lacking. Of note, we found that TCM CPGs tend to present more recommendations with strongly, and there is a lack of against recommendations. Another notable finding of our study is that more strong recommend recommendations in TCM CPGs are based on low or very low certainty of evidence, but a considerable proportion of discordant recommendations do not provide reasonable justification for this questionable behavior.

### Strength and limitations

To our knowledge, this is the first methodological study to compare GRADE CPGs and TCM CPGs in terms of the influencing factors of recommendations. Our study has several strengths. First, we conducted a systematic and comprehensive retrieval, screening, data extraction and analysis of GRADE CPGs and TCM CPGs, respectively. Second, we included the CPGs published in 2018–2022, which contributed to a comprehensive assess of the methodology of GRADE CPGs and TCM CPGs in recent years. Third, we compared the GRADE CPGs with the TCM CPGs to help clarify the deficiencies and improvement of the TCM CPGs.

We acknowledged that one limitation of our study is that we did not further assess the reliability of certainty of evidence judgments reported in the CPGs, therefore, it is remains possible that some low or very low certainty of evidence was classified as medium or high certainty of evidence, and medium or high certainty of evidence was classified as low or very low certainty of evidence, so there may be partially recommendations actually discordant recommendations and some discordant recommendations actually may not be truly discordant. Meanwhile, this study analyzed the randomly selected CPGs for all durations, so there could be bias. In addition, our results were not compared with other CPGs such as Korean, Japan or other countries, which may limit the generalization of the results.

### Comparison with prior work

A prior study used the Appraisal of Guidelines Research and Evaluation II instrument (AGREE II) to evaluate the guidelines for the symptomatic management of fever in children published in 2011 – 2016, but it focused on the overall quality of the guidelines [27]. An Australian guideline methodological study showed an inconsistency between guideline development that claims to use the GRADE approaches and the true GRADE approaches, however, it included guidelines published in 2011 – 2018 [28]. Colby et al. found that about one-third of the United States organizations used GRADE in developing evidence-based guidelines, but compliance using the GRADE domain was not optimistic and its findings were consistent with ours [29]. Those three studies lack attention to the comprehensive consideration of influencing factors in the formation of evidence to recommendations, and the link between the certainty of evidence and the strength of recommendations had not yet further been fully explored.

### Implications

TCM CPGs have limited consideration of the influencing factors in the formation of recommendations, and lack of comprehensive consideration such as resource requirements, health equity, acceptability, etc., which may be one of the reasons for presenting a large number of unreasonable recommendations. In addition, some guidelines claim to apply the GRADE approaches, but we cannot prove that they did implement them, perhaps due to a misunderstanding of the GRADE approaches or adherence to the GRADE approaches but not explicitly reported. With the increasing apply of GRADE approaches in the development of guidelines, the advantages become increasingly apparent, Hence it is extremely important to advocate the correct application of GRADE approaches and continue to improve the adherence and further training of the methodology.

Careful consideration is needed. There are great differences between the diagnosis and treatment of TCM and western medicine. The evaluation of TCM effect has the difficulties caused by the discipline characteristics. When adopting GRADE approach in guideline development, some important effect impactors were not considered, such as the adequacy of the individualized treatments. Besides this, lacking of evidence for resource use, health equity, acceptability and feasibility decreased the applicability of GRADE approaches in the development of TCM guidelines. However, this is what TCM researchers need to work on rather than considering this as problems of the GRADE approaches. At present, the research in this field is still immature, and further exploration is needed in the future. We think this provides additional rational in future researches.

## Conclusions

The TCM CPGs lack a comprehensive consideration of multiple influencing determinants from evidence to recommendations, and they lack the application of GRADE approaches. Four key determinants—desirable anticipated effects, undesirable anticipated effects, balance between desirable and undesirable effects, certainty of the evidence- was better considered and have more evidence to support, but other factors such as values and preferences, resource requirements, feasibility, health equity, problem priority is lack of consideration. This may be caused by the irrational use of the GRADE approaches. TCM CPGs present a considerable number of inappropriate discordant recommendations–strong recommendations based on low or very low certainty of evidence but cannot provide reasonable justification. Attention should be paid to appropriate GRADE approaches use in the future.

### Supplementary Information

**Additional file 1: Appendix A.** Search strategy.

**Additional file 2: Appendix B.** Standardization of the certainty of the evidence and the strength of the recommendation.

**Additional file 3: Appendix C.** Included clinical practice guidelines.

## Data Availability

The datasets used or analysed during the current study are available from the corresponding author on reasonable request.
