# Supplementary material for: Comprehensive consideration of multiple determinants from evidence to recommendations in guidelines for most traditional Chinese medicine was suboptimal: a systematic review
Source: BMC Complement Med Ther. 2024 Jan 4;24:19. doi: 10.1186/s12906-023-04321-0 (PMC10765706; doi:10.1186/s12906-023-04321-0)
Supplement: Supplementary file 1 — Additional file 1: Appendix A. Search strategy. [file 12906_2023_4321_MOESM1_ESM.docx]

## Appendix A

**Search Strategy**

### Database search of the Traditional Chinese Medicine clinical practice guidelines

**Retrieved Date:2023.02.08**

| **Database** | **Step** | **Search Strategy** | **References** |
| --- | --- | --- | --- |
| PubMed | #1 | "Chinese patent medicine"[Title/Abstract] OR "proprietary Chinese medicine"[Title/Abstract] OR "massage"[Title/Abstract] OR "tuina"[Title/Abstract] OR "Acupuncture"[MeSH Terms] OR "Acupuncture Therapy"[MeSH Terms] OR "acupotomy"[Title/Abstract] OR "cupping"[Title/Abstract] OR "traditional Chinese medicine"[Title/Abstract] | 71680 |
|  | #2 | (("Practice Guideline" [Publication Type] OR "Guideline" [Publication Type] OR "Guidelines as Topic"[Mesh]) OR ((((((("clinical guidance"[Title/Abstract]) OR ("clinical practice guidance"[Title/Abstract])) OR ("guideline*"[Title/Abstract])) OR ("guidance*"[Title/Abstract])) OR ("clinical guidance"[Title/Abstract])) OR ("clinical guideline"[Title/Abstract])) OR ("evidence-based guideline"[Title/Abstract]))) | 703708 |
|  | #3 | (("Chinese patent medicine"[Title/Abstract] OR "proprietary Chinese medicine"[Title/Abstract] OR ("massage"[Title/Abstract] OR "tuina"[Title/Abstract] OR ("Acupuncture"[MeSH Terms] OR "Acupuncture Therapy"[MeSH Terms] OR "acupotomy"[Title/Abstract] OR "cupping"[Title/Abstract] OR "traditional Chinese medicine"[Title/Abstract]))) AND ("Practice Guideline"[Publication Type] OR "Guideline"[Publication Type] OR "Guidelines as Topic"[MeSH Terms] OR ("clinical guidance"[Title/Abstract] OR "clinical practice guidance"[Title/Abstract] OR "guideline*"[Title/Abstract] OR "guidance*"[Title/Abstract] OR "clinical guidance"[Title/Abstract] OR "clinical guideline"[Title/Abstract] OR "evidence-based guideline"[Title/Abstract]))) AND (2018/1/1:2022/12/31[pdat]) | 1115 |
| Embase | #1 | ('acupuncture'/exp OR acupunct*:ti,ab,kw OR moxibust*:ti,ab,kw OR acupotomy:ti,ab,kw OR cupping:ti,ab,kw OR massage:ti,ab,kw OR tuina:ti,ab,kw OR 'Chinese patent medicine':ti,ab,kw OR 'Chinese patent drug':ti,ab,kw OR 'proprietary Chinese medicine':ti,ab,kw OR 'proprietary Chinese drug':ti,ab,kw OR 'traditional Chinese medicine':ti,ab,kw) | 113304 |
|  | #2 | 'clinical practice guidance':ti,ab,kw OR 'guideline*':ti,ab,kw OR 'guidance*':ti,ab,kw OR 'clinical guidance':ti,ab,kw OR 'clinical guideline':ti,ab,kw OR 'evidence-based guideline':ti,ab,kw OR 'practice guideline'/exp | 1267342 |
|  | #3 | ('acupuncture'/exp OR acupunct*:ti,ab,kw OR moxibust*:ti,ab,kw OR acupotomy:ti,ab,kw OR cupping:ti,ab,kw OR massage:ti,ab,kw OR tuina:ti,ab,kw OR 'Chinese patent medicine':ti,ab,kw OR 'Chinese patent drug':ti,ab,kw OR 'proprietary Chinese medicine':ti,ab,kw OR 'proprietary Chinese drug':ti,ab,kw OR 'traditional Chinese medicine':ti,ab,kw) AND ('clinical practice guidance':ti,ab,kw OR 'guideline*':ti,ab,kw OR 'guidance*':ti,ab,kw OR 'clinical guidance':ti,ab,kw OR 'clinical guideline':ti,ab,kw OR 'evidence-based guideline':ti,ab,kw OR 'practice guideline'/exp) AND (2018:py OR 2019:py OR 2020:py OR 2021:py OR 2022:py) | 2796 |

We also searched four Chinese databases (China National Knowledge Infrastructure, VIP Database for Chinese Technical Periodicals, Wanfang, and Chinese Biomedical Literature Database), the Chinese database search strategy can be obtained by contacting us.

### Database search of the GRADE clinical practice guidelines

**Retrieved Date:2023.02.08**

| **Database** | **Step** | **Search Strategy** | **References** |
| --- | --- | --- | --- |
| PubMed | #1 | (((((((((("GRADE Centre"[Title/Abstract]) OR("GRADE Centre"[Author])) OR ("GRADE Center"[Author])) OR ("GRADE working group"[Title/Abstract])) OR ("GRADE working group"[Author])) OR ("McMaster University GRADE Centre"[Title/Abstract])) OR ("McMaster University GRADE Centre"[Author])) OR (Gordon H. Guyatt[Author])) OR (Holger J. Schünemann[Author])) OR ("McMaster University GRADE Center"[Author])) OR ("McMaster University GRADE Center"[Title/Abstract]) | 1395 |
|  | #2 | (("Practice Guideline" [Publication Type] OR "Guideline" [Publication Type] OR "Guidelines as Topic"[Mesh]) OR ((((((("clinical guidance"[Title/Abstract]) OR ("clinical practice guidance"[Title/Abstract])) OR ("guideline*"[Title/Abstract])) OR ("guidance*"[Title/Abstract])) OR ("clinical guidance"[Title/Abstract])) OR ("clinical guideline"[Title/Abstract])) OR ("evidence-based guideline"[Title/Abstract]))) | 702974 |
|  | #3 | (("GRADE Centre"[Title/Abstract] OR "GRADE Centre"[Author] OR "GRADE Center"[Author] OR "GRADE working group"[Title/Abstract] OR "GRADE working group"[Author] OR "McMaster University GRADE Centre"[Title/Abstract] OR "McMaster University GRADE Centre"[Author] OR guyatt, gordon h[Author] OR (schunemann, holger j[Author] OR j schunemann, holger[Author]) OR "McMaster University GRADE Center"[Author]) AND ("Practice Guideline"[Publication Type] OR "Guideline"[Publication Type] OR "Guidelines as Topic"[MeSH Terms] OR ("clinical guidance"[Title/Abstract] OR "clinical practice guidance"[Title/Abstract] OR "guideline*"[Title/Abstract] OR "guidance*"[Title/Abstract] OR "clinical guidance"[Title/Abstract] OR "clinical guideline"[Title/Abstract] OR "evidence-based guideline"[Title/Abstract])) AND 2018/01/01:2022/12/31[Date - Publication]) AND (2018/1/1:2022/12/31[pdat]) | 275 |
| Embase | #1 | 'grade centre':ti,ab,kw OR 'grade centre':au OR 'grade center':au OR 'grade working group':ti,ab,kw OR 'grade working group':au OR 'mcmaster university grade centre':ti,ab,kw OR 'mcmaster university grade centre':au OR 'gordon h. guyatt':au OR 'holger j. schünemann':au OR 'mcmaster university grade center':ti,ab,kw OR 'mcmaster university grade center':au | 408 |
|  | #2 | 'clinical practice guidance':ti,ab,kw OR 'guideline*':ti,ab,kw OR 'guidance*':ti,ab,kw OR 'clinical guidance':ti,ab,kw OR 'clinical guideline':ti,ab,kw OR 'evidence-based guideline':ti,ab,kw OR 'practice guideline'/exp | 1266459 |
|  | #3 | ('grade centre':ti,ab,kw OR 'grade centre':au OR 'grade center':au OR 'grade working group':ti,ab,kw OR 'grade working group':au OR 'mcmaster university grade centre':ti,ab,kw OR 'mcmaster university grade centre':au OR 'gordon h. guyatt':au OR 'holger j. schünemann':au OR 'mcmaster university grade center':ti,ab,kw OR 'mcmaster university grade center':au) AND ('clinical practice guidance':ti,ab,kw OR 'guideline*':ti,ab,kw OR 'guidance*':ti,ab,kw OR 'clinical guidance':ti,ab,kw OR 'clinical guideline':ti,ab,kw OR 'evidence-based guideline':ti,ab,kw OR 'practice guideline'/exp) AND (2018:py OR 2019:py OR 2020:py OR 2021:py OR 2022:py) | 117 |

We also searched four Chinese databases (China National Knowledge Infrastructure, VIP Database for Chinese Technical Periodicals, Wanfang, and Chinese Biomedical Literature Database), the Chinese database search strategy can be obtained by contacting us.
