# Supplementary material for: Comprehensive consideration of multiple determinants from evidence to recommendations in guidelines for most traditional Chinese medicine was suboptimal: a systematic review
Source: BMC Complement Med Ther. 2024 Jan 4;24:19. doi: 10.1186/s12906-023-04321-0 (PMC10765706; doi:10.1186/s12906-023-04321-0)
Supplement: Supplementary file 2 — Additional file 2: Appendix B. Standardization of the certainty of the evidence and the strength of the recommendation. [file 12906_2023_4321_MOESM2_ESM.docx]

## Appendix B

### Standardization of the certainty of the evidence and the strength of the recommendation

**Standardization of the certainty of the evidence**

| \| Level of evidence \| Explanation \| \| --- \| --- \| \| I \| Large sample, randomized studies, clear results, false positives or false negatives with very low errors \| \| II \| Small sample, randomized study, inconclusive results, false positives and / or false negatives errors are high \| \| III \| Non-randomized, concurrent controlled study and expert consensus based on ancient literature \| \| IV \| Non-randomized, historical control, or contemporary expert consensus \| \| V \| Case reports, non-controlled study and expert opinion \| |
| --- | --- | --- | --- | --- | --- | --- | --- | --- | --- | --- | --- | --- |
| \| level of evidence \| Explanation \| \| --- \| --- \| \| Ⅰa \| The body of evidence consisting of at least different types of studies in randomized controlled trials, cohort studies, case-control studies, case series studies, and the effects of different studies are consistent \| \| Ⅰb \| A single randomized controlled trial with adequate confidence \| \| Ⅱa \| Semi-randomized controlled trial or a cohort study \| \| Ⅱb \| Case – control study; \| \| Ⅲa \| Case series of historic controls; \| \| Ⅲb \| Case series of Self-control before and after； \| \| Ⅳ \| It has been widely used in clinical case reports and historical records； \| \| Ⅴ \| Expert views and clinical experience without systematic research and verification, as well as case reports and historical records that have not been widely used in clinical practice for a long time \| |
| \| Level of evidence \| Explanation \| \| --- \| --- \| \| 1a \| Systematic evaluation of a homogeneous RCT \| \| 1b \| Individual RCT (narrow confidence interval) \| \| 1c \| Full or no disease case series \| \| 2a \| Systematic evaluation of the homogeneous cohort studies \| \| 2b \| Single cohort study (including low quality RCT, if follow-up rate <80%) \| \| 2c \| Outcome study, ecological studies \| \| 3a \| Systematic evaluation of homogeneous case-control studies \| \| 3b \| Individual case controls \| \| 4 \| Case series studies (including low-quality cohort and case-control studies) \| \| 5 \| \| Expert opinion based on experience without rigorous demonstration \| \| --- \| \| |
| \| Level of evidence \| Explanation \| \| --- \| --- \| \| A \| Data were obtained from multiple randomized clinical trials or meta-analyses \| \| B \| Data were obtained from a single randomized clinical trial or multiple non-randomized controlled studies \| \| C \| Only for expert consensus opinion and (or) small-scale studies, retrospective studies, registered studies \| |

### Standardization of the strength of the recommendation

| \| Strength \| Explanation \| \| --- \| --- \| \| A \| At least one randomized controlled clinical trial is required as part of the high quality and coherent literature (evidence from levels 1a and 1b) \| \| B \| Good clinical studies need to be done relevant to the subject, but no randomized controlled trials (evidence from a, b and grade) \| \| C \| Reporting or comments and / or clinical trials but lack direct high quality clinical studies (evidence from and level V) \| |
| --- | --- | --- | --- | --- | --- | --- | --- | --- |
| \| Strength \| Explanation \| \| --- \| --- \| \| A \| Results were consistent for level 1 studies \| \| B \| Extrapolation of grades 2 or 3 studies with consistent results or of grade 1 studies \| \| C \| Level 4 studies, or extrapolation of level 2 or 3 studies \| \| D \| 5 levels of evidence, or any level of conflicting or inconclusive research \| |
| \| Strength \| Explanation \| \| --- \| --- \| \| strong \| The recommended treatment benefits far outweigh the side effects \| \| weak \| The recommended treatments are uncertain, with high side effects or antagonism \| |
| Principles of recommendation intensity: if the number of people recommended for a treatment measure exceeds 75% of the total; if the proportion not recommended is 50%, it is not recommended; other cases are weak recommendation. |
| \| Strength \| Explanation \| \| --- \| --- \| \| I \| Means a proven and (or) consistently recognized beneficial, useful, and effective procedure or treatment, recommended \| \| II \| An operation or treatment with useful (valid) evidence \| \| IIa \| The evidence (opinion) tends to be useful / effective and the application of these procedures or treatments is justified \| \| IIb \| The relevant evidence (opinion) can not be fully proved useful / effective and can be considered for application \| \| III \| Any operation or treatment that is proven and (or) uniformly considered useless and (or) ineffective and potentially harmful in some cases is not recommended \| |
