# Supplementary material for: Comprehensive consideration of multiple determinants from evidence to recommendations in guidelines for most traditional Chinese medicine was suboptimal: a systematic review
Source: BMC Complement Med Ther. 2024 Jan 4;24:19. doi: 10.1186/s12906-023-04321-0 (PMC10765706; doi:10.1186/s12906-023-04321-0)
Supplement: Supplementary file 3 — Additional file 3: Appendix C. Included clinical practice guidelines. [file 12906_2023_4321_MOESM3_ESM.docx]

## Appendix C

### Included clinical practice guidelines

| **Number** | **Title of clinical practice guideline** | **Country/region** | **Developer** | **Year** |
| --- | --- | --- | --- | --- |
|  |  |  |  |  |
| 1 | Medical cannabis or cannabinoids for chronic pain: a clinical practice guideline | International group | BMJ | 2021 |
| 2 | A living WHO guideline on drugs to prevent covid-19 | World Health Organization | BMJ | 2021 |
| 3 | SGLT-2 inhibitors or GLP-1 receptor agonists for adults with type 2 diabetes: a clinical practice guideline | International group | BMJ | 2021 |
| 4 | Colorectal cancer screening with faecal immunochemical testing, sigmoidoscopy or colonoscopy: a clinical practice guideline | International group | BMJ | 2019 |
| 5 | Atraumatic (pencil-point) versus conventional needles for lumbar puncture: a clinical practice guideline | International group | BMJ | 2018 |
| 6 | Antibiotics after incision and drainage for uncomplicated skin abscesses: a clinical practice guideline | International group | BMJ | 2018 |
| 7 | Patent foramen ovale closure, antiplatelet therapy or anticoagulation therapy alone for management of cryptogenic stroke? A clinical practice guideline | International group | BMJ | 2018 |
| 8 | Gastrointestinal bleeding prophylaxis for critically ill patients: a clinical practice guideline | International group | BMJ | 2020 |
| 9 | Corticosteroid therapy for sepsis: a clinical practice guideline | International group | BMJ | 2018 |
| 10 | Prostate cancer screening with prostate-specific antigen (PSA) test: a clinical practice guideline | International group | BMJ | 2018 |
| 11 | 2021 American College of Rheumatology/Vasculitis Foundation Guideline for the Management of Polyarteritis Nodosa | America | American College of Rheumatology/Vasculitis  Foundation | 2021 |
| 12 | 2021 American College of Rheumatology/Vasculitis Foundation Guideline for the Management of Antineutrophil Cytoplasmic Antibody–Associated Vasculitis | America | American College of Rheumatology/Vasculitis  Foundation | 2021 |
| 13 | 2021 American College of Rheumatology/Vasculitis Foundation Guideline for the Management of Giant Cell Arteritis and Takayasu Arteritis | America | American College of Rheumatology/Vasculitis  Foundation | 2021 |
| 14 | 2020 American College of Rheumatology Guideline for the Management of Gout | America | American College of Rheumatology | 2020 |
| 15 | 2020 American College of Rheumatology Guideline for the Management of Reproductive Health in Rheumatic and Musculoskeletal Diseases | America | American College of Rheumatology | 2020 |
| 16 | 2019 American College of Rheumatology/Arthritis Foundation Guideline for the Management of Osteoarthritis of the Hand, Hip, and Knee | America | American College of Rheumatology/Vasculitis  Foundation | 2021 |
| 17 | ASH, ABHH, ACHO, Grupo CAHT, Grupo CLAHT, SAH, SBHH, SHU, SOCHIHEM, SOMETH, Sociedad Paname~na de Hematolog ıa, SPH, and SVH 2021 guidelines for management of venous thromboembolism in Latin America | America | American Society of Hematology | 2021 |
| 18 | American Society of Hematology living guidelines on the use of anticoagulation for thromboprophylaxis for patients with COVID-19: March 2022 update on the use of anticoagulation in critically ill patients | America | American Society of Hematology | 2021 |
| 19 | Evidence-based guidelines for supportive care of patients with Ebola virus disease | / | / | 2018 |
| 20 | ASH ISTH NHF WFH 2021 guidelines on the management of von Willebrand disease | America | American Society of Hematology （ASH）、The International Society for Thrombosis and Hemostasis （ISTH）、The National Hemophilia Foundation （NHF） and World Federation of Hemophilia（WFH） | 2021 |
| 21 | European guidelines on breast cancer screening and diagnosis | Europe | European Commission | 2020 |
| 22 | American Society of Hematology 2020 guidelines for treating newly diagnosed acute myeloid leukemia in older adults | America | American Society of Hematology | 2020 |
| 23 | American Society of Hematology 2020 guidelines for management of venous thromboembolism: treatment of deep vein thrombosis and pulmonary embolism | America | American Society of Hematology | 2020 |
| 24 | Transfusion strategies in non-bleeding critically ill adults: a clinical practice guideline  from the European Society of Intensive Care Medicine | Europe | European Society of Intensive Care  Medicine | 2020 |
| 25 | American Society of Hematology 2019 guidelines for management of venous thromboembolism: prevention of venous thromboembolism in surgical hospitalized patients | America | American Society of Hematology | 2020 |
| 26 | 2018 American College of Rheumatology/National Psoriasis Foundation Guideline for the Treatment of Psoriatic Arthritis | America | American College of Rheumatology | 2018 |
| 27 | American Society of Hematology 2018 guidelines for management of venous thromboembolism: diagnosis of venous thromboembolism | America | American Society of Hematology | 2018 |
| 28 | American Society of Hematology 2018 guidelines for management of venous thromboembolism: prophylaxis for hospitalized and nonhospitalized medical patients | America | American Society of Hematology | 2018 |
| 29 | American Society of Hematology 2018 Guidelines for management of venous thromboembolism: treatment of pediatric venous thromboembolism | America | American Society of Hematology | 2018 |
| 30 | American Society of Hematology 2018 guidelines for management of  venous thromboembolism: venous thromboembolism in the context  of pregnancy | America | American Society of Hematology | 2018 |
| 31 | American Society of Hematology 2018 guidelines for management of venous thromboembolism: optimal management of anticoagulation therapy | America | American Society of Hematology | 2018 |
| 32 | American Society of Hematology 2018 guidelines for management of venous thromboembolism: heparin-induced thrombocytopenia | America | American Society of Hematology | 2018 |
| 33 | Treatment of patients with nonsevere and severe coronavirus disease 2019: an evidence based guideline | China | / | 2019 |
| 34 | Subacromial decompression surgery for adults with shoulder pain: a clinical practice guideline | International group | BMJ | 2019 |
| 35 | Oxygen therapy for acutely ill medical patients: a clinical practice guideline | International group | BMJ | 2018 |
| 36 | Plasma exchange and glucocorticoid dosing for patients with ANCA-associated vasculitis: a clinical practice guideline | International group | BMJ | 2022 |
| 37 | Remdesivir for severe covid-19: a clinical practice guideline | International group | BMJ | 2020 |
| 38 | Dual antiplatelet therapy with aspirin and clopidogrel for acute high risk transient ischaemic attack and minor ischaemic stroke: a clinical practice guideline | International group | BMJ | 2018 |
| 39 | 2021 American College of Rheumatology/Vasculitis Foundation Guideline for the Management of Kawasaki Disease | America | American College of Rheumatology/Vasculitis  Foundation | 2021 |
| 40 | Endovascular thrombectomy and intravenous alteplase in patients with acute ischemic stroke due to large vessel occlusion: A clinical practice guideline | China | BMJ | 2022 |
| 41 | Guidelines for the prevention and management of children and adolescents with COVID‑19 | China | / | 2022 |
| 42 | Joint 2022 European Society of Thoracic Surgeons and The American Association for Thoracic Surgery guidelines for the prevention of cancer-associated venous thromboembolism in thoracic surgery | Europe | European Society of Thoracic Surgeons and The American  Association | 2022 |
| 43 | Canadian Rheumatology Association Recommendation for the Use of COVID-19 Vaccination for Patients With Autoimmune Rheumatic Diseases | Canada | Canadian Rheumatology Association | 2021 |
| 44 | American Society of Hematology, ABHH, ACHO, Grupo CAHT, Grupo CLAHT, SAH, SBHH, SHU, SOCHIHEM, SOMETH, Sociedad Paname~ na de Hematolog ıa, Sociedad Peruana de Hematolog ıa, and SVH 2022 guidelines for prevention of venous thromboembolism in surgical and medical patients and long-distance travelers in Latin America | America | American Society of Hematology | 2022 |
| 45 | World Allergy Organization (WAO) Diagnosis and Rationale for Action against Cow’s Milk  Allergy (DRACMA) Guideline update – XIV – Recommendations on CMA immunotherapy | International group | World Allergy Organization | 2022 |
| 46 | Guidelines for the diagnosis and treatment of integrated traditional Chinese and Western medicine for early-onset ovarian insufficiency | China | Chinese Association of Integrative Medicine | 2022 |
| 47 | Guidelines for the diagnosis and treatment of integrated traditional Chinese and Western medicine for tubal pregnancy | China | Chinese Association of Integrative Medicine | 2021 |
| 48 | Guidelines for the diagnosis and treatment of integrated traditional Chinese and Western medicine for abnormal uterine bleeding | China | Chinese Association of Integrative Medicine | 2020 |
| 49 | Guidelines for the diagnosis and treatment of integrated traditional Chinese and Western medicine in endometriosis | China | Chinese Association of Integrative Medicine | 2019 |
| 50 | Clinical practice guideline of integrated traditional Chinese and western medicine:atlantoaxial dislocation(AAD)(2019) | China | Chinese Association of Integrative Medicine | 2019 |
| 51 | Guidelines for the diagnosis and treatment of integrated traditional Chinese medicine and western medicine in osteoporosis | China | Chinese Association of Integrative Medicine | 2019 |
| 52 | Guidelines for diagnosis and treatment of integrated Chinese and western Medicine (Trial Edition) | China | Chinese Association of Integrative Medicine | 2018 |
| 53 | Diabetic foot disease combined with traditional Chinese medicine disease syndrome diagnosis and treatment guidelines | China | World federation of Chinese medicine societies, Doctor Society of integrative Medicine, Chinese Medical Doctor Association. | 2021 |
| 54 | Diabetic peripheral neuropathy syndrome combined with the diagnosis and treatment guidelines | China | World federation of Chinese medicine societies, Doctor Society of integrative Medicine, Chinese Medical Doctor Association. | 2021 |
| 55 | Diabetic cerebrovascular disease syndrome combined with the diagnosis and treatment guidelines | China | World federation of Chinese medicine societies, Doctor Society of integrative Medicine, Chinese Medical Doctor Association. | 2022 |
| 56 | Diabetic cardiomyopathy syndrome combined with the diagnosis and treatment guidelines | China | World federation of Chinese medicine societies, Doctor Society of integrative Medicine, Chinese Medical Doctor Association. | 2022 |
| 57 | Diabetic retinopathy syndrome combined with the diagnosis and treatment guidelines | China | World federation of Chinese medicine societies, Doctor Society of integrative Medicine, Chinese Medical Doctor Association. | 2021 |
| 58 | Diabetic nephropathy syndrome combined with the diagnosis and treatment guidelines | China | World federation of Chinese medicine societies, Doctor Society of integrative Medicine, Chinese Medical Doctor Association. | 2021 |
| 59 | Prediabetes disease syndrome combined with the diagnosis and treatment guidelines | China | World federation of Chinese medicine societies, Doctor Society of integrative Medicine, Chinese Medical Doctor Association. | 2021 |
| 60 | Guidelines for combining the diagnosis and treatment of diabetes mellitus combined with hyperlipidemia | China | World federation of Chinese medicine societies, Doctor Society of integrative Medicine, Chinese Medical Doctor Association. | 2021 |
| 61 | Hyperthyroidism syndrome combined with the diagnosis and treatment guidelines | China | World federation of Chinese medicine societies, Doctor Society of integrative Medicine, Chinese Medical Doctor Association. | 2021 |
| 62 | Guidelines for combining the diagnosis and treatment of hyperuricemia and gout disease syndrome | China | World federation of Chinese medicine societies, Doctor Society of integrative Medicine, Chinese Medical Doctor Association. | 2021 |
| 63 | Type 2 diabetes disease syndrome combined with the diagnosis and treatment guidelines | China | World federation of Chinese medicine societies, Doctor Society of integrative Medicine, Chinese Medical Doctor Association. | 2021 |
| 64 | Guidelines for the integration of traditional Chinese and Western medicine for the diagnosis and treatment of acute myocardial infarction | China | World federation of Chinese medicine societies, Doctor Society of integrative Medicine, Chinese Medical Doctor Association. | 2018 |
| 65 | International clinical practice guidelines for traditional Chinese medicine for age-related macular degeneration | China | World federation of Chinese medicine societies | 2022 |
| 66 | International traditional Chinese medicine clinical practice guide for glycoipid metabolism | China | World federation of Chinese medicine societies | 2022 |
| 67 | International traditional Chinese medicine clinical practice guidelines for retinal vein occlusion | China | World federation of Chinese medicine societies | 2022 |
| 68 | Guidelines for clinical diagnosis and treatment of gastric stomach (gastrotosis) | China | World federation of Chinese medicine societies | 2020 |
| 69 | International clinical practice guideline of Chinese medicine chronic obstructive pulmonary disease | China | World federation of Chinese medicine societies | 2020 |
| 70 | Guidelines for integrated traditional Chinese and Western medicine diagnosis and treatment of angina pectoris after coronary revascularization | China | World federation of Chinese medicine societies | 2020 |
| 71 | The clinical practice guidelines of traditional Chinese medicine for distention and fullness (2018) | China | World federation of Chinese medicine societies | 2019 |
| 72 | International traditional Chinese medicine clinical practice guidelines leak out | China | World federation of Chinese medicine societies | 2021 |
| 73 | International clinical practice guide of traditional Chinese medicine, menstruation early | China | World federation of Chinese medicine societies | 2021 |
| 74 | International traditional Chinese medicine clinical practice guidelines for degenerative lumbar spinal stenosis | China | World federation of Chinese medicine societies | 2021 |
| 75 | International traditional Chinese medicine clinical practice guidelines for rheumatoid arthritis | China | World federation of Chinese medicine societies | 2020 |
| 76 | Clinical practice guidelines of traditional Chinese medicine for oral sores | China | World federation of Chinese medicine societies | 2020 |
| 77 | International traditional Chinese medicine clinical practice guidelines for atopic dermatitis | China | World federation of Chinese medicine societies | 2021 |
| 78 | The international traditional Chinese medicine clinical practice guide for forgetfulness | China | World federation of Chinese medicine societies | 2019 |
| 79 | Guidelines for clinical application of traditional Chinese medicine in dry eye (2021) | China | China Association of Traditional Chinese Medicine | 2022 |
| 80 | Guidelines for the clinical application of proprietary Chinese patent medicine in the treatment of idiopathic rare and weak sperm male infertility | China | China Association of Traditional Chinese Medicine | 2022 |
| 81 | Guidelines for clinical application of proprietary Chinese medicine for breast hyperplasia (2021 Edition) | China | China Association of Traditional Chinese Medicine | 2022 |
| 82 | Guidelines for the clinical application of proprietary Chinese medicine in the treatment of essential hypertension | China | China Association of Traditional Chinese Medicine | 2022 |
| 83 | Guidelines for the clinical application of proprietary Chinese medicine for treating chronic prostatitis | China | China Association of Traditional Chinese Medicine | 2022 |
| 84 | Guidelines for the clinical application of proprietary Chinese patent medicine in the treatment of osteoporosis | China | China Association of Traditional Chinese Medicine | 2022 |
| 85 | Guidelines for the clinical application of proprietary Chinese medicine in the adjuvant treatment of hyperthyroidism (Graves disease) | China | China Association of Traditional Chinese Medicine | 2022 |
| 86 | Guidelines for the clinical application of proprietary Chinese medicine in the treatment of vascular dementia | China | China Association of Traditional Chinese Medicine | 2021 |
| 87 | Guidelines for the clinical application of proprietary Chinese patent medicine for neonatal jaundice | China | China Association of Traditional Chinese Medicine | 2021 |
| 88 | Guidelines for clinical application of proprietary Chinese medicine for knee osteoarthritis (2020) | China | China Association of Traditional Chinese Medicine | 2020 |
| 89 | Guidelines for the clinical application of proprietary Chinese patent medicine for eczema treatment | China | China Association of Traditional Chinese Medicine | 2021 |
| 90 | Guidelines for the clinical application of proprietary Chinese patent medicine for the treatment of age-related macular degeneration (wet) | China | China Association of Traditional Chinese Medicine | 2021 |
| 91 | Guidelines for the clinical application of Chinese patent medicine for chronic kidney disease stage 3-5 (non-dialysis) | China | China Association of Traditional Chinese Medicine | 2021 |
| 92 | Guidelines for the clinical application of proprietary Chinese medicine for treating coronary heart disease | China | China Association of Traditional Chinese Medicine | 2021 |
| 93 | Guidelines for the clinical application of proprietary Chinese medicine for the treatment of menopausal syndrome | China | China Association of Traditional Chinese Medicine | 2021 |
| 94 | Clinical practice guidelines of traditional Chinese medicine rehabilitation for incomplete paraplegia | China | other societies | 2021 |
| 95 | Tuina for children with acute diarrhea: an evidence-based clinical guideline | China | other societies | 2021 |
| 96 | Clinical Practice Guide for knee osteoarthritis | China | other societies | 2020 |
| 97 | Guidelines for traditional Chinese medicine Diagnosis and Treatment of Knee Osteoarthritis (2020 edition) | China | other societies | 2020 |
| 98 | Clinical practice guidelines in traditional Chinese medicine rehabilitation—rheumatoid arthritis | China | other societies | 2020 |
| 99 | Clinical practice guidelines for knee osteoarthritis in integrated traditional Chinese and western medicine | China | other societies | 2021 |
| 100 | Guidelines for the multidisciplinary diagnosis and treatment of integrated Traditional Chinese and Western medicine for chronic prostatitis | China | other societies | 2020 |
| 101 | Editorial explanation for clinical practice guideline for stable chronic obstructive pulmonary disease with traditional Chinese medicine | China | other societies | 2020 |
| 102 | Instructions for complication of guidelines for clinical practice of traditional Chinese medicine in migraine | China | other societies | 2020 |
| 103 | Clinical practice guidelines in modified Tai Chi therapy for post-stoke dyskinesia | China | other societies | 2022 |
| 104 | Clinical practice guidelines for stroke of Chinese medicine rehabilitation | China | other societies | 2019 |
| 105 | Guidelines for diagnosis and treatment of osteoarthritis based  on combination of disease and syndrome | China | China Association of Chinese Medicine | 2019 |
| 106 | Clinical guidelines for diagnosis and treatment of knee osteoarthritis(Xibibing) in orthopedics and traumatology of traditional Chinese medicine | China | China Association of Chinese Medicine | 2019 |
| 107 | Guidelines for clinical diagnosis and treatment of pediatrics in traditional Chinese medicine: Granial disease (pediatric hydrocephalus) | China | China Association of Chinese Medicine | 2020 |
| 108 | Guidelines for traditional Chinese medicine diagnosis and treatment of community-acquired pneumonia | China | China Association of Chinese Medicine | 2018 |
| 109 | Guideline for Traditional Chinese Medicine intervention  program after routine treatment of western medicine for Ⅰ-  Ⅲ stage colorectal cancer | China | China Association of Chinese Medicine | 2022 |
| 110 | Intervention program for prevention and treatment of asthma | China | China Association of Chinese Medicine | 2022 |
| 111 | Preventive intervention program for chronic obstructive pulmonary disease | China | China Association of Chinese Medicine | 2022 |
| 112 | Clinical diagnosis and treatment guidelines of mental diseases for integrated Chinese and western medicine -- tic disorder | China | China Association of Chinese Medicine | 2021 |
| 113 | Traditional Chinese medicine treatment guidelines on coronary heart disease before and after percutaneous coronary intervention | China | China Association of Chinese Medicine | 2018 |
| 114 | Rheumatoid arthritis syndrome combined with the diagnosis and treatment guidelines | China | China Association of Chinese Medicine | 2018 |
| 115 | International clinical practice guideline of traditional Chinese medicine ‧ viral myocarditis | China | World federation of Chinese medicine societies | 2020 |
| 116 | Guidelines for TCM Diagnosis and Treatment of acute pancreatitis, a common disease of the digestive system (primary doctor edition) | China | China Association of Chinese Medicine | 2020 |
| 117 | Guidelines for diagnosis and treatment of high altitude polycythemia in traditional Chinese medicine | China | China Association of Chinese Medicine | 2021 |
| 118 | Clinical guidelines for diagnosis and treatment of frozen shoulder in traditional Chinese medicine | China | China Association of Chinese Medicine | 2022 |
| 119 | Chinese Medicine Guideline for Diagnosis and Treatment of Osteoporosis Fracture | China | China Association of Chinese Medicine | 2022 |
| 120 | Diagnosis and treatment guideline for Chinese medicine on  acute trachea-bronchitis | China | China Association of Chinese Medicine | 2021 |
| 121 | Guidelines for the multidisciplinary diagnosis and treatment of integrated Traditional Chinese and Western medicine for benign prostatic hyperplasia | China | other societies | 2022 |
| 122 | Acupuncture for cancer pain:  an evidence-based clinical practice guideline | China | other societies | 2022 |
| 123 | Acupuncture for low back pain: a clinical practice guideline from the  Hong Kong taskforce of standardized acupuncture practice | China | other societies | 2021 |
| 124 | Clinical practice guidelines for the treatment of allergic  rhinitis in children with traditional Chinese medicine | China | other societies | 2021 |
| 125 | Evidence-based guidelines of clinical practice wIth acupuncture and moxibustion Pain after ankle sprain | China | China Association for Acupuncture and Moxibustion | 2019 |
| 126 | Evidence-based guidelines of clinical practice with acupuncture and moxibustion Tenosynovitis pain | China | China Association for Acupuncture and Moxibustion | 2019 |
| 127 | Evidence-based guidelines of clinical practice with acupuncture and moxibustion Swelling and pain of eyes | China | China Association for Acupuncture and Moxibustion | 2019 |
| 128 | Evidence-based guidelines of clinical practice with acupuncture and moxibustion Piman | China | China Association for Acupuncture and Moxibustion | 2019 |
| 129 | Evidence-based guidelines of clinical practice with acupuncture and moxibustion Postoperative urinary retention | China | China Association for Acupuncture and Moxibustion | 2019 |
| 130 | Evidence-based guidelines of clinical practice with acupuncture and moxibustion Toothache | China | China Association for Acupuncture and Moxibustion | 2019 |
| 131 | Evidence-based guidelines of clinical practice with acupuncture and moxibustion Hypochondriac pain | China | China Association for Acupuncture and Moxibustion | 2019 |
| 132 | Evidence-based guidelines of clinical practice with Acupuncture and moxibustion is tending pain caused by lower extremity varicose veins | China | China Association for Acupuncture and Moxibustion | 2019 |
| 133 | Evidence-based clinical practice guidelines for Acupuncture-Sciatica | China | China Association for Acupuncture and Moxibustion | 2019 |
